# A Law to Regulate the Practice of Medicine

**Published:** 1895-10

**Authors:** 


### A Law to Regulate the Practice of Medicine.

The medical profession of Ohio is moving in the direction of securing efficient legislation to regulate the practice of medicine. First and foremost to secure for the people immunity against the abominations of quackery, and the enormous evils it brings upon the people; and second to give encouragement to legitimate medicine, and to secure a higher and more advanced medical education, to secure greater thoroughness and efficiency in its practice. Ohio practically has no law regulating medical practice, and as a result quackery has grown to gigantic proportions. The quacks of the State doubtless obtain nearly, if not quite, as much money as all the legitimate physicians of the State together; and for what do the people give this immense amount of money? Simply for the satisfaction of being gulled, deceived and too often for having health injured or destroyed, and many a time death resulting.

Ohio has a law establishing and maintaining a Board of Health, whose duty it is to attend to the sanitary and health condition of the people of the State, and in the discharge of its duty vigorous measures are employed, and rightly, too. But how shall we account for the course of our Legislature in the establishment and support of such an agency, while at the same time there is an absolute refusal, so far as the past is concerned, to do anything to abolish or even check a practice that produces more suffering, disease and indirectly, if not directly, death than any other agency, except the liquor traffic? What have our Legislators been thinking about? The people of the State being robbed of their money, health and too often of life itself, by this evil, and no effort even attempted for its removal or curtailment.

The press of the State, both secular and religious, with very few exceptions, lend themselves to the maintainnance of this enormous iniquity; they are in a very large degree responsible for it; without their aid it would be crippled and shorn of a large part of its power. There are but few publications in Ohio the advertising pages of which should not make an honest man's face tingle and burn with shame. The explanation of this state of things is found in the greed of the proprietors of our papers. Brethren of the press, you had better right up on this question.

It is to be hoped that the incoming Legislature will see this question in its true light and recognize its enormity, and take righteous action in the matter.

And now a word to the dentists of the State: you are a part or branch of the medical profession and should be interested in everything that pertains to its welfare and prosperity, and on this account it is entirely proper that the dentists of the State make common cause with the physicians in the securing of needed legislation. Consult with the physician of your respective localities and learn in what way you can aid in promoting this cause, and in the attainment of success you will be the instrument of bringing great good to the community.

The law regulating the practice of dentistry needs a little readjustment, and it will be wise to secure all the proper aid possible to that end.

It is to be hoped that the men who are sent to the Legislature this fall will view some things at least in a different light from their predecessors.

